# A study of college students' intention to use metaverse technology for basketball learning based on UTAUT2

**DOI:** 10.1016/j.heliyon.2022.e10562

**Published:** 2022-09-08

**Authors:** Fangfang Yang, Longfei Ren, Chao Gu

**Affiliations:** a Department of Sports Science of Honam University, Gwangju, 62399, South Korea; b Department of Culture and Arts Management of Honam University, Gwangju, 62399, South Korea

**Keywords:** Metaverse, Basketball teaching, Virtual space, UTAUT2

## Abstract

Recent advancements in virtual reality technology have attracted increasing attention from enterprises and scholars, and many new related products have been launched. Due to the current COVID-19 epidemic, the non-face-to-face teaching environment will seriously affect students' basketball learning. We therefore combined basketball learning with metaverse technology, discussed basketball teaching in a virtual reality environment, and examined the influencing factors of college students' intentions to use metaverse technology. In the light of UTAUT2, a new research model was proposed, and quantitative research was carried out. The results of a survey of 1074 valid samples revealed that habits and attitudes are crucial factors in the success of basketball learning using a metaverse. The findings also indicate that grade and gender are moderator variables.

## 1 Introduction

### 1.1 Research background

As a result of the long-term impact of the epidemic, the global culture has become non-face-to-face. In order to overcome the limitations of time and space, we have seen cultural diffusion made possible by digital technology (J.-E. Jeon, 2021). Due to the large number of students and teachers who have been affected by the outbreak, online learning formats are becoming increasingly popular (Sukendro et al., 2020). Approximately ten times the total number of university students enrolled in at least one online course (Smith et al., 2008). During the outbreak, the Politecnico di Torino in Italy ceased lectures and offered 600 virtual courses a day for its students as well as online courses for more than 16,000 individuals (Favale et al., 2020). Using VR and AR technology in the natural science classroom of the third grade of primary school can enhance students' learning efficiency and concentration, while VR technology can satisfy students' curiosity (Anggara et al., 2021). It may be possible to combine traditional education with distance education after the COVID-19 pandemic (Borukova and Kuleva, 2020).

People's lives cannot be separated from information technologies, and the field of education is also affected by information technologies, and e-learning can enhance teaching quality and promote creativity among students (Hikmawan et al., 2019). It has been shown that students who use interactive electronic content modules are better able to perform math calculations and retain information than those who use traditional teaching methods (Prabakaran & Saravanakumar, 2020). E-learning may be the trend of the future, since it is convenient for everyone, allows users to adjust learning time to their schedules, provides access to updated learning content at any time, and maximizes the potential of students (Radha et al., 2020). The educational electronic platform can help teachers develop students' learning motivation, create tasks, educational projects and problems for the students, and encourage the students to combine STEAM subject information to develop their own skills and abilities (Soroko et al., 2020). The combination of e-learning and artificial intelligence promotes individualized learning for students and engages students in the best learning experiences (Mansor et al., 2020). The Second Life virtual reality learning tool has entered the classroom as a means of cultivating multidisciplinary situational cognition, action learning and experiential learning in schools. It is widely recognized for its ability to be used in teaching and its potential for future research (Wang and Burton, 2013). The Mateverse (VoRetx) platform allows users to create virtual worlds. Users can explore the world of mateverse and communicate with people (virtual characters). There are different social activities that can simulate real-world activities in the metaworld. Through the use of a virtual 3D environment, students are able to interact with their teachers in real time (Jovanović & Milosavljević, 2022).

Due to the continuous development of the metaverse topic, a growing number of scholars are studying the technology associated with the metaverse. In comparison with traditional basketball education, metaverse basketball overcomes several limitations, including the limitation of space. Online courses can benefit from its authentic engagement experience by offering an interactive communication experience like traditional courses. Virtual video game worlds provide a sense of fun that facilitates learning. Learning resources can be mobilized quickly through the internet, and the format and participation experience can be more engaging than traditional boring methods of instruction. It differs from sports video games by focusing more on real body movements and real-time virtual interaction as opposed to simple joysticks or buttons. Even though traditional teaching methods still dominate the PE curriculum, they clearly fail to meet the learning needs of students. It examines the integration of metaverse technology into physical education in this article. A feasibility study is being conducted to assess whether the metaverse can be utilized in basketball curriculum and to determine students' intention to use the technology. This study examines the feasibility of incorporating metaverse technology into sports basketball courses and may serve as a guide for other scholars interested in combining metaverse technology into sports.

There is a large amount of evidence that in the context of a COVID-19 pandemic, the online teaching mode should be combined to speed up teaching progress. Metaverse technology has a positive effect on student learning. Through the use of metaverse technology, students will be immersed in a virtual environment, combined with VR, AR and other technologies, as if they were there, improving students' sense of participation in reality and increasing students' sense of reality for online basketball learning. Combining metaverse technology and sports basketball lessons due to technical movements, tactical complexity, and partner collaboration. With the help of metaverse technology, students are able to learn basketball techniques, tactics, and referee knowledge via a virtual environment, in combination with teachers' scientific real-time guidance and analysis. Based on the collected data, the most likely method of calculation was used to analyze the data. Additionally, it provides suggestions regarding the use of metaverse technology within the field of sports, which can facilitate the teaching and learning of sports courses.

### 1.2 Research motivation and purpose

Many people have been restricted in their activities, and many schools have reduced contact through online courses due to the effects of the COVID-19. It is essential to assist students to achieve better learning outcomes in online courses in light of the adverse effects of the COVID-19. It is worthwhile to use metaverse technology for online teaching in the current environment, and it is imperative to improve the metaverse functions for educational purposes. This study examined the factors that influence Chinese college students' willingness to use Metaverse technology for online basketball courses in conjunction with UTAUT2. As well as providing some design recommendations for the future development of metaverse technology in physical education.

## 2 Literature review

### 2.1 Metaverse

A metaverse first appeared in Neal Stephenson's 1992 novel *Snowstorm* (Joshua, 2017). "Meta" means transcendence, "verse" in the cosmic stem of "universe", which represents users communicating with each other and the application of 3D software (Duan et al., 2021). The number of individuals using online platforms has increased dramatically in recent years, especially during the epidemic (Sweeney, 2019). The definition of virtual heritage as Champion defines it: combines the intangible and tangible, emphasizing the importance of interaction and immersion (Champion, 2016). Considering Huggett's definition of virtual legacy, he points out that presence and authenticity are crucial for people to experience virtual reality (Huggett, 2020). In the metaverse, participants can use their identities in the virtual space to interact with other participants in real time (Ng et al., 2021).

A virtual community that includes real people will be created using network and computing technology, artificial intelligence technology, blockchain technology, Internet of Things technology, and video game technology (Kim and Jeon, 2021). It is possible to create new things in the virtual world, but the limited connection between the real world and the virtual world affects its functionality (Lee et al., 2021, Lee et al., 2021). Human desires and ideas may be realized in a three-dimensional virtual environment (Kwon, 2021). In the metaverse, there are features such as identity, friends, immersion, anywhere, diversity, low latency, economics, and civilization (Ning et al., 2021). A virtual augmented physical reality is merged with a persistent virtual space in order to establish a barrier-free connection between the virtual world and the physical one (Bolger, 2021). Meatworld can be divided into two distinct areas: the first is the desire for humans to exist in a completely virtual environment which is better than reality to some extent; the second, there is the technology that can finally link the real world to the virtual one (van der Merwe, 2021). Combining metaverse technology with learning, participants can feel the presence of learning, but learning emphasizes the interaction of participants and the educational environment (J. Jeon and Jung, 2021).

### 2.2 E-learning

Combining traditional teaching methods with cloud-based e-learning to meet the evolving learning needs of students, especially in higher education (Lew et al., 2019). E-learning refers to a method of learning that utilizes the Internet in order to improve student independence and enhance student-centered learning (Schworm and Gruber, 2012). E-learning can be used in a variety of settings, however the teaching methods must be adapted to the environment (Burgos et al., 2007). Incorporating higher education with metaverse technology, virtualizing and enhancing reality, integrating simulation technology with blockchain technology, and cultivating high-level technical talent needed in the industry (Damar, 2021). Metaverse provides a safe and efficient environment for both education and business, through virtual metaverse technology, participants can continue to learn and expand their experiential learning opportunities (Kim and Jeon, 2021). Using e-learning in English courses can improve students' grammar and writing skills, reduce their fear of communicating in English, and increase their willingness to speak out and share their opinions (Hortal, 2021). E-learning refers to the application and process of online learning, computer-based learning, distance learning, and dissemination of information through the Internet, external networks, intranets, audio, video, and satellite television (Abdulazeez and Zeebaree, 2018).

E-Learning is the most effective way to encourage students to continue learning without compromising their performance (Almanthari et al., 2020). Online learning is currently an important component of educational institutions during the epidemic period, and an effective method of teaching (Radha et al., 2020). E-learning facilitates the learning of both students and teachers, and a variety of e-learning and online education methods have been used to advance education through the use of e-learning (Mahalakshmi and Radha, 2020). As a result of COVID-19, e-learning is the only way to continue education, a very important educational tool, and an important tool for HR training and institutional training (I. A. Khan, 2020). Today, ICT is used in classrooms around the world, and participants who use e-learning devices demonstrate a higher level of cognitive ability (Moletsane and Venter, 2018). In higher education institutions, e-learning is increasingly being combined with face-to-face instruction (Estriegana et al., 2019).

### 2.3 Basketball instructions

The purpose of basketball instruction is to teach students how to play basketball and how to referee basketball under the guidance of an educator and a student (Aufan et al., 2019). Similarly, the physical education curriculum undergoes continuous improvement as the times evolve. With physical education teachers combining e-learning with basketball teaching, using online learning media, the learning quality of basketball physical education classes and the teaching quality of the teachers will be improved (Sopy and Hasibuan, 2020). Basketball learning application media programs greatly improve students' fundamental skills in dribbling, passing, shooting, and layups (Riza et al., 2021). Combining game activities with basketball instruction will promote the learning of basketball skills in junior high school students, as well as improve their technical skills and ability to perform on an individual basis (Hamidi, 2018). College students learn basketball tactics along with programmed e-learning to develop their tactical thinking and decision-making skills (El-Saleh, 2020). A basketball course should implement a variety of teaching methods and use electronic programs and computer tools in order to teach basketball skills and improve the enthusiasm of students (Karmajouna et al., 2021).

### 2.4 UTAUT2

As a result of expanding the UTAUT model in 2012, three new constructs were introduced, namely hedonic motivation, price value, and habit, whereas gender, age, and experience were set as moderators, which were widely used in the following years (Venkatesh et al., 2012). UTAUT has been validated as a very effective model for predicting the intention of college teachers to use blended learning (García, del Dujo and Rodríguez, 2014). In the study of smart mobile devices, the UTAUT2 model can well explain and analyze users' technology acceptance behaviors (Huang and Kao, 2015).

#### 2.4.1 Performance expectancy

An individual's performance expectancy refers to their degree of confidence that the system they are using will be beneficial to them (Venkatesh et al., 2003). In other words, it is the degree to which participants are able to accomplish tasks with the assistance of technology (Venkatesh et al., 2012). Students' performance expectancy refers to how well mobile learning services facilitate their learning activities (Al-Hujran et al., 2014). The performance expectancy measures the degree to which students believe mobile learning can enhance their academic performance (Chao, 2019). Providing users with a high level of performance expectancy can increase their willingness to use Internet banking (Rahi et al., 2019). A health integration system in the medical field improves user performance, work efficiency, and quality of health care, which increases participants' willingness to use the system (Jahanbakhsh et al., 2018). Thus, it can be assumed that:

H1 In college students who are learning basketball using the Metaverse, performance expectancy has a positive effect on behavior intention.

H1a In college students who are learning basketball using the Metaverse, performance expectancy has a positive effect on use behavior.

H1b In college students who are learning basketball using the Metaverse, performance expectancy has a positive effect on attitude.

H1c College students' attitudes regarding performance expectancy using metaverse technology to learn basketball are not moderated by gender.

H1d College students' attitudes regarding performance expectancy using metaverse technology to learn basketball are not moderated by grade.

#### 2.4.2 Effort expectancy

Effort expectations refer to how easy it will be for the user to use the new system (Venkatesh et al., 2003). In older adults, effort expectancy reflects their comfort and ease in using ICT (Macedo, 2017). The effort expectancy is defined as the ease with which students adopt mobile learning services (Al-Hujran et al., 2014). Due to the specific knowledge and skills required to use mobile banking technology, effort expectancy plays a critical role in the customer's intention to use mobile banking technology (Alalwan et al., 2016). In the survey of learners' willingness to use mobile learning, it was found that learners' social needs have a positive impact on their effort expectations (Thongsri et al., 2018). Therefore, it is reasonable to assume that:

H2 In college students who are learning basketball using the metaverse, effort expectancy has a positive effect on behavioral intention.

H2a In college students who are learning basketball using the metaverse, effort expectancy has a positive effect on use behavior.

H2b In college students who are learning basketball using the metaverse, effort expectancy has a positive effect on attitude.

#### 2.4.3 Social influence

The term "social influence" refers to the degree of influence that surrounding people have on an individual when they use the system (Venkatesh et al., 2003). In the context of mobile learning, social influence refers to the extent to which students think they should utilize mobile learning services that are important to them, such as those offered by their teachers, family, friends, etc (Al-Hujran et al., 2014). In developing countries, social influence promotes the adoption of mobile government services at the initial adoption stage (Ahmad and Khalid, 2017). Participant willingness to use e-government services can be influenced by social influence (Kurfalı et al., 2017). It can be seen from the above literature that social influence plays an important role in determining participants' behavioral intention, which has already been demonstrated in the previous literature. Thus, it can be assumed that:

H3 In college students who are learning basketball using the metaverse, social influence has a positive effect on behavioral intention.

H3a In college students who are learning basketball using the metaverse, social influence has a positive effect on use behavior.

H3b In college students who are learning basketball using the metaverse, social influence has a positive effect on attitude.

#### 2.4.4 Facilitating conditions

In general, facilitator conditions refer to the degree of confidence an individual has that devices and technologies exist and are available for use (Venkatesh et al., 2003). Students' attitudes towards the resources and support they will receive as a result of adopting mobile learning services can be described as facilitating conditions (Al-Hujran et al., 2014). Thus, it can be assumed that:

H4 In college students who are learning basketball using the metaverse, facilitating conditions has a positive effect on behavioral intention.

H4a In college students who are learning basketball using the metaverse, facilitating conditions has a positive effect on use behavior.

H4b In college students who are learning basketball using the metaverse, facilitating conditions has a positive effect on attitude.

H4c College students' attitudes regarding facilitating conditions using metaverse technology to learn basketball are moderated by gender.

H4d College students' attitudes regarding facilitating conditions using metaverse technology to learn basketball are moderated by grade.

#### 2.4.5 Hedonic motivation

An individual's feeling of happiness when he or she uses a new technology is known as hedonic motivation (Brown and Venkatesh, 2005). Consumer purchase intent can be influenced by hedonic motivation for products displayed in social media advertisements. Marketers will design more innovative and creative advertising, increasing its intrinsic effectiveness and interactivity, and promoting consumers' hedonic motivation (Alalwan, 2018). In contrast to chatbots, augmented reality apps are more likely to provide an enjoyable user experience (Moriuchi et al., 2021). The hedonic motivation provides strong evidence that online banking users are more likely to use online banking, such as the hedonic motivation, which has a significant impact on the willingness of online banking users to utilize online banking (S Rahi et al., 2018). The behavioral intention of the participant to use the new technology in a positive manner (Alalwan et al., 2017). The purpose of Hedonic motivation is to provide participants with a sense of self-fulfillment. By using the new system, participants are able to have fun (Ramírez-Correa, Rondán-Cataluña, Arenas-Gaitán, & Martín-Velicia, 2019). Thus, it can be assumed that:

H5 In college students who are learning basketball using the metaverse, hedonic motivation has a positive effect on behavioral intention.

H5a In college students who are learning basketball using the metaverse, hedonic motivation has a positive effect on use behavior.

H5b In college students who are learning basketball using the metaverse, hedonic motivation has a positive effect on attitude.

H5c College students' attitudes regarding hedonic motivation using metaverse technology to learn basketball are moderated by gender.

H5d College students' attitudes regarding hedonic motivation using metaverse technology to learn basketball are moderated by grade.

#### 2.4.6 Habit

The term habit refers to an individual's active, progressively automated behavior for learning (Limayem et al., 2007). Habits are determined by the level of interaction and familiarity with a technology, and they develop at different levels over time (Venkatesh et al., 2012). Consumers' smartphone usage of habit has a significant impact on its perceived value and ease of use (Hubert et al., 2017). Habit is the most important factor influencing the willingness of accounting students to use mobile learning in higher education (Moorthy, Yee, T'ing, & Kumaran, 2019). The most important factor contributing to people's continued use of mobile applications is habit (Tam et al., 2020). The most important factor for people to use online games is habit (Ramírez-Correa et al. (2019)). Thus, it can be assumed that:

H6 In college students who are learning basketball using the metaverse, habit has a positive effect on behavioral intention.

H6a In college students who are learning basketball using the metaverse, habit has a positive effect on use behavior.

H6b In college students who are learning basketball using the metaverse, habit has a positive effect on attitude.

H6c College students' habit regarding behavioral intention using metaverse technology to learn basketball are moderated by gender.

H6d College students' habit regarding behavioral intention using metaverse technology to learn basketball are moderated by grade.

#### 2.4.7 Behavioural intention

Behavioural Intention relates to the impact of participants' adoption of e-government services on their behavior (Kurfalı et al., 2017). Behavioral intention refers to the degree to which an individual is consciously planning his or her future behavior (Ramírez-Correa et al., 2019). Individuals will rely on their own perceptions of usefulness and ease of use to form their intentions, and intentions can predict the level of acceptance of technology in the future (Szajna, 1996). The positive attitude that students demonstrate when using the e-learning system contributes to their behaviour intention (Salloum et al., 2019). Students' behavioural intentions with regard to mobile learning are greatly affected by social influences (Briz-Ponce et al., 2017) People's behavior intention is influenced by their habits when using new technologies in cross-cultural contexts (Merhi et al., 2019; Nikolopoulou et al., 2020). Thus, it can be assumed that:

H7 In college students who are learning basketball using the metaverse, behavioural intention has a positive effect on use behavior.

H7a College students' behavioural intention regarding use behavior using metaverse technology to learn basketball are not moderated by gender.

H7b College students' behavioural intention regarding use behavior using metaverse technology to learn basketball are not moderated by grade.

#### 2.4.8 Use behavior

The number of times an individual uses information technology is referred to as use behavior (Ramírez-Correa et al., 2019). There is evidence that the cultural dimension, collectivism, and uncertainty avoidance have significant moderating effects on the use behavior of customers engaged in online banking (I. U. Khan, Hameed and Khan, 2017).

### 2.5 Attitude

The focus of psychology and related disciplines is on attitudes, and attitude changes lead to changes in behavior (Howe and Krosnick, 2017). There is a significant role for attitude in influencing emotional risk perception and behavioural intention (Bae and Chang, 2021). By utilizing VR experience technology, a positive attitude will encourage tourists to visit (Tussyadiah, Wang, Jung, & Tom Dieck, 2018). Attitudes are intrinsically subjective and the level of certainty associated with them reflects an individual's confidence and belief in the attitude (Tormala and Rucker, 2018). Attitudes toward brands are influenced by consumer perceptions of social influence (Yang et al., 2017). Therefore, it can be assumed that:

H8 In college students who are learning basketball using the metaverse, attitude has a positive effect on behavioral intention.

H8a In college students who are learning basketball using the metaverse, attitude has a positive effect on use behavior.

H8b College students' attitude regarding behavioural intention using metaverse technology to learn basketball are not moderated by gender and grade.

H8c College students' attitude regarding use behavior using metaverse technology to learn basketball are not moderated by gender and grade.

## 3 Research method

In this study, we conducted an online survey of participants who had similar experience in interactive teaching through the Internet. In this study, there is no actual interactive teaching for the subjects in offline occasions, so the human-computer interaction process and medical-related ethical issues is not involved. Therefore, there is not applicable for Institutional review board statement.

### 3.1 Research subject

COVID-19 pandemic has seriously impacted the progress of physical education, resulting in insufficient exercise for students and limited teaching effectiveness, which has presented new challenges to online instruction. A new concept of the metaverse and the development of tactile devices bring new ideas to the teaching of cyber basketball. Promoting and implementing this technology will mark an important turning point in the reform of physical education and sports.

This study examines the intention of college students to use metaverse technology in basketball instruction. The data was analyzed using structural equation modeling in order to explore the relationship between the factors. The questionnaire has been revised to include a related video resource, which explains the concept of the metaverse, current basketball-related metaverse resources, and future possibilities for using metaverse technology in basketball education. In this study, we examine students' intention to use metaverse technology for college basketball courses by examining the experiences of freshman and sophomore students in basketball courses.

Firstly, the age and knowledge base of college students enable them to evaluate the application of new technologies with a greater degree of objectivity. Additionally, general basketball classes are only offered in the first and second grades, as these students have higher learning needs than their counterparts at other levels. Since the COVID-19 pandemic, most Chinese college students have accepted long-term online courses, which allows them to gain a wealth of experience in the introduction of new technologies. Therefore, we chose them as the subject of this study.

### 3.2 Questionnaire design

Based on the original UTAUT2 questionnaire, we modified and adapted the questions according to the theme of this article, according to college students' willingness to utilize metaverse basketball teaching, and adding attitude factors. According to Table 1, the reference source, code, item, and source information for the latent variable are as follows:

**Table 1:** Measurement scale.

Latent Variable	Coding	Item	Source
Performance Expectancy	PE1	The metaverse was helpful to me in learning basketball.	[(Venkatesh et al., 2012)]
	PE2	The metaverse has helped me to learn basketball more quickly.	
	PE3	By utilizing the metaverse, I can improve my basketball learning efficiency.	
Effort Expectancy	EE1	For me, learning how to use metaverse technology for basketball learning is easy.	
	EE2	In my experience, the metaverse technology used to learn basketball has been clear and understandable.	
	EE3	Basketball learning with metaverse technology was easy to use for me.	
	EE4	With the help of metaverse technology, I have been able to learn basketball proficiently.	
Social Influence	SI1	According to someone close to me, I should use metaverse technology for learning basketball skills.	
	SI2	There are individuals who influence my behavior who recommend that I use metaverse technology for basketball learning.	
	SI3	The people I value prefer me to play basketball using metaverse technology.	
Facilitating Conditions	FC1	In the metaverse, I have access to the resources necessary to learn basketball.	
	FC2	In the metaverse, I have all the information I need to learn about basketball.	
	FC3	Using the metaverse technology to learn basketball is compatible with other technology I use.	
	FC4	I can get assistance from others if I am having trouble with my metaverse learning techniques.	
Hedonic Motivation	HM1	Learning basketball with metaverse technology is a fun experience.	
	HM2	Learning basketball through metaverse technology is an enjoyable experience.	
	HM3	Learning basketball using metaverse technology is a rewarding experience.	
Habit	HT1	Learning basketball using metaverse technology will be my kind of habit.	
	HT2	I am addicted to learning basketball through the use of metaverse technology.	
	HT3	It is necessary for me to use metaverse technology in order to learn basketball.	
Behavioral Intention	BI1	Future basketball learning will continue to utilize metaverse technology.	
	BI2	As part of my daily life, I will always seek to use metaverse technology as a means of learning basketball.	
	BI3	In order to improve my basketball skills, I plan to continue to use metaverse technology on a regular basis.	[(S. M. Lee and Lee, 2020)]
use behavior	AC1	It is a pleasure to use metaverse technology to learn basketball	
	AC2	For the purpose of learning basketball, I will actively use metaverse technology	
	AC3	It would be my pleasure to recommend basketball learning with metaverse technology to others.	
	AC4	As a basketball student, I am confident in my ability to use metaverse technology.	
Attitude	AT1	As a basketball learner, I have a positive opinion of the Metaverse if given the opportunity to do so.	[(Q. Jiang, J. Sun, C. Yang, & C. Gu, 2022)]
	AT2	Using metaverse technology for basketball learning can provide valuable services.	
	AT3	Learning basketball with Metaverse technology can be a rewarding experience	

### 3.3 Data collection

The questionnaires were collected via online responses from January to February 2022. Participants who clicked on the link to the survey to view the description of the survey responded voluntarily and were fully informed of the survey.

In total, 1,774 samples were collected in this study, of which 1,074 were valid. The recovery efficiency rate was 65.54 percent. In this study, there are 34 questionnaire questions and 1074 valid samples, and the statistics are based on these samples.

In this study, there are a total of 1074 freshmen and sophomores selected from the eastern, western, central, northeastern, Hong Kong, Macao, and Taiwan regions of China. 415 of them are male, 659 are female, 636 are freshmen, and 438 are sophomores, representing seven disciplines such as humanities and engineering technology. Table 2 shows the results.

**Table 2:** Basic information of interviewees.

Sample	Category	Number	Percentage
Gender	Male	415	38.6
	Female	659	61.4
Grade	Freshman	636	59.2
	Sophomore	438	40.8
Major	Natural science	118	11.0
	Engineering and Technology	262	24.4
	Medicine and health Sciences	149	13.9
	Agricultural Science	62	5.8
	Social sciences	188	17.5
	Humanities	182	16.9
	Science of physical culture and sports	113	10.5
Hometown	Eastern Region	298	27.7
	Middle Region	423	39.4
	western region	316	29.4
	Northeast Region	36	3.4
	Hong Kong, Macao and Taiwan regions	1	0.1

## 4 Result

### 4.1 Hypothesis test of data distribution

This study assumes normality and linearity for the distribution of the returned questionnaires. Hypothesis testing will be conducted on the returned questionnaires. Based on the skewness and kurtosis of each facet, the results of the test of the normality of the distribution indicate that the absolute value of skewness is between 0.520 (HT) and 0.856 (PE), and the absolute value of kurtosis is 0.003. Between (HT) and 0.995 (PE), the absolute value of skewness is less than 3.0, and the absolute value of kurtosis is less than 8.0. The distribution of the data collected by the formal test conforms to the univariate normal distribution (Kline, 2015).

### 4.2 Reliability analysis

It is the Cronbach's Alpha coefficient and the modified total correlation coefficient (CITC) that are used to validate the data in this study. In Table 3, it is evident that the CITC of all facets is greater than 0.5, and the reliability coefficient does not significantly improve after the question is deleted. The Cronbach's reliability coefficient is greater than 0.7. As a result, the questionnaires and scales used in this study are internally consistent.

**Table 3:** Reliability analysis.

Item	Corrected Item Total Correlation	Cronbach's Alpha If Item Deleted	Cronbach's Alpha	Item	Corrected Item Total Correlation	Cronbach's Alpha If Item Deleted	Cronbach's Alpha
HT1	.593	.749	.787	PE1	.739	.775	.851
HT2	.648	.687		PE2	.688	.824	
HT3	.648	.689		PE3	.737	.777	
HM1	.655	.753	.813	AT1	.661	.713	.801
HM2	.644	.764		AT2	.631	.744	
HM3	.693	.714		AT3	.647	.727	
FC1	.739	.776	.845	BI1	.692	.735	.820
FC2	.690	.799		BI2	.673	.754	
FC3	.676	.805		BI3	.658	.768	
FC4	.621	.828		UB1	.736	.798	.855
SI1	.678	.722	.811	UB2	.695	.815	
SI2	.640	.761		UB3	.677	.823	
SI3	.662	.738		UB4	.678	.823	
EE1	.727	.806	.857				
EE2	.697	.818					
EE3	.680	.826					
EE4	.696	.819					

### 4.3 Exploratory factor analysis

In this article, we use SPSS 26.0 to conduct an exploratory factor analysis in order to verify one facet of each factor. As shown in Table 4, the KMO values of all constructs are greater than 0.7, and the importance of the Bartlett sphericity test is less than 0.05, thus an exploratory factor analysis can be carried out (Norusis, 1992). The commonality of each construct item is greater than 0.5, indicating good alignment (Hair et al., 1998). The factor loading is greater than 0.6, and only one new factor with an eigenvalue greater than 1 can be identified, indicating that the constructs are of high quality.

**Table 4:** Exploratory factor analysis results.

Construct	KMO	Bartlett's Sphere Test	Item	Commonality	Factor Loading	Eigenvalue	Total Variation Explained
HT	.702	.000	HT1	.663	.814	2.107	70.244%
			HT2	.722	.850		
			HT3	.723	.850		
HM	.713	.000	HM1	.719	.848	2.185	72.845%
			HM2	.707	.841		
			HM3	.759	.871		
FC	.808	.000	FC1	.749	.865	2.729	68.228%
			FC2	.692	.832		
			FC3	.677	.823		
			FC4	.611	.782		
SI	.714	.000	SI1	.744	.863	2.176	72.544%
			SI2	.704	.839		
			SI3	.728	.853		
EE	.827	.000	EE1	.731	.855	2.798	69.952%
			EE2	.697	.835		
			EE3	.676	.822		
			EE4	.695	.834		
PE	.727	.000	PE1	.790	.889	2.314	77.122%
			PE2	.736	.858		
			PE3	.788	.887		
AT	.711	.000	AT1	.731	.855	2.146	71.542%
			AT2	.699	.836		
			AT3	.717	.847		
BI	.718	.000	BI1	.754	.868	2.208	73.590%
			BI2	.734	.857		
			BI3	.719	.848		
UB	.822	.000	UB1	.742	.862	2.786	69.642%
			UB2	.696	.834		
			UB3	.674	.821		
			UB4	.674	.821		

### 4.4 Confirmatory factor analysis

#### 4.4.1 Convergence validity test

In this article, a confirmatory factor analysis is performed using AMOS V22.0 to analyze a structural equation model. As is evident from Table 5, the standardized factor loading is greater than 0.6 in this study, the SMC is greater than 0.4, the t is greater than 2.58, the sig is significant, the reliability of the study structure component is greater than 0.7, and the mean variance extraction (AVE) is greater than 0.5, which suggests that this construct has good converging validity (Fornell and Larcker, 1981a).

**Table 5:** Convergence validity test.

	Factor loading	SMC	t	Sig.	S.E.	CR	AVE
HT1	.725	.526	25.481	.002	.022	.789	.555
HT2	.766	.586	27.391	.001	.020		
HT3	.743	.553	26.334	.001	.019		
HM1	.751	.564	27.110	.001	.025	.815	.595
HM2	.750	.562	27.053	.001	.020		
HM3	.811	.657	30.138	.001	.017		
FC1	.815	.664	31.175	.001	.016	.846	.580
FC2	.772	.595	28.788	.001	.017		
FC3	.764	.583	28.369	.002	.018		
FC4	.691	.478	24.726	.002	.026		
SI1	.795	.632	29.456	.002	.019	.810	.588
SI2	.717	.514	25.571	.001	.025		
SI3	.786	.619	29.014	.001	.018		
EE1	.787	.619	29.591	.002	.018	.857	.600
EE2	.791	.625	29.810	.001	.015		
EE3	.752	.565	27.729	.002	.019		
EE4	.767	.589	28.536	.001	.019		
PE1	.837	.701	31.953	.001	.016	.853	.659
PE2	.773	.598	28.537	.001	.018		
PE3	.823	.678	31.173	.001	.016		
AT1	.755	.570	26.993	.001	.024	.801	.573
AT2	.756	.572	27.053	.001	.020		
AT3	.760	.577	27.223	.001	.022		
BI1	.797	.636	29.868	.001	.017	.821	.604
BI2	.785	.616	29.212	.001	.018		
BI3	.749	.561	27.360	.001	.021		
UB1	.804	.646	30.500	.001	.015	.855	.597
UB2	.782	.612	29.328	.001	.016		
UB3	.752	.565	27.711	.001	.019		
UB4	.750	.562	27.598	.001	.020		

#### 4.4.2 Discriminant validity test

A pairwise correlation analysis was performed on each aspect of this study. According to Tables 6 and 7, the HTMT value is less than 0.85, and the square root of AVE for each aspect is greater than the correlation coefficient between the structures, indicating that each aspect of this study has good discriminant validity (Fornell and Larcker, 1981a).

**Table 6:** Discriminant validity test.

	HT	HM	FC	SI	EE	PE	AT	BI	UB
HT	.745								
HM	.452	.771							
FC	.561	.628	.762						
SI	.507	.592	.625	.767					
EE	.488	.593	.660	.632	.775				
PE	.378	.519	.509	.579	.613	.812			
AT	.448	.545	.563	.479	.479	.453	.757		
BI	.638	.530	.614	.538	.543	.444	.593	.777	
UB	.593	.556	.594	.527	.534	.452	.612	.668	.773

**Table 7:** Result of the heterotrait-monotrait ratio test.

	HT	HM	FC	SI	EE	PE	AT	BI	UB
HT									
HM	.573								
FC	.690	.759							
SI	.640	.730	.755						
EE	.602	.710	.775	.759					
PE	.468	.624	.601	.697	.718				
AT	.568	.675	.686	.594	.578	.547			
BI	.793	.649	.735	.659	.648	.531	.732		
UB	.725	.666	.700	.633	.624	.529	.740	.798	

### 4.5 Model validation

#### 4.5.1 Common method deviation model analysis

**Figure 1:**
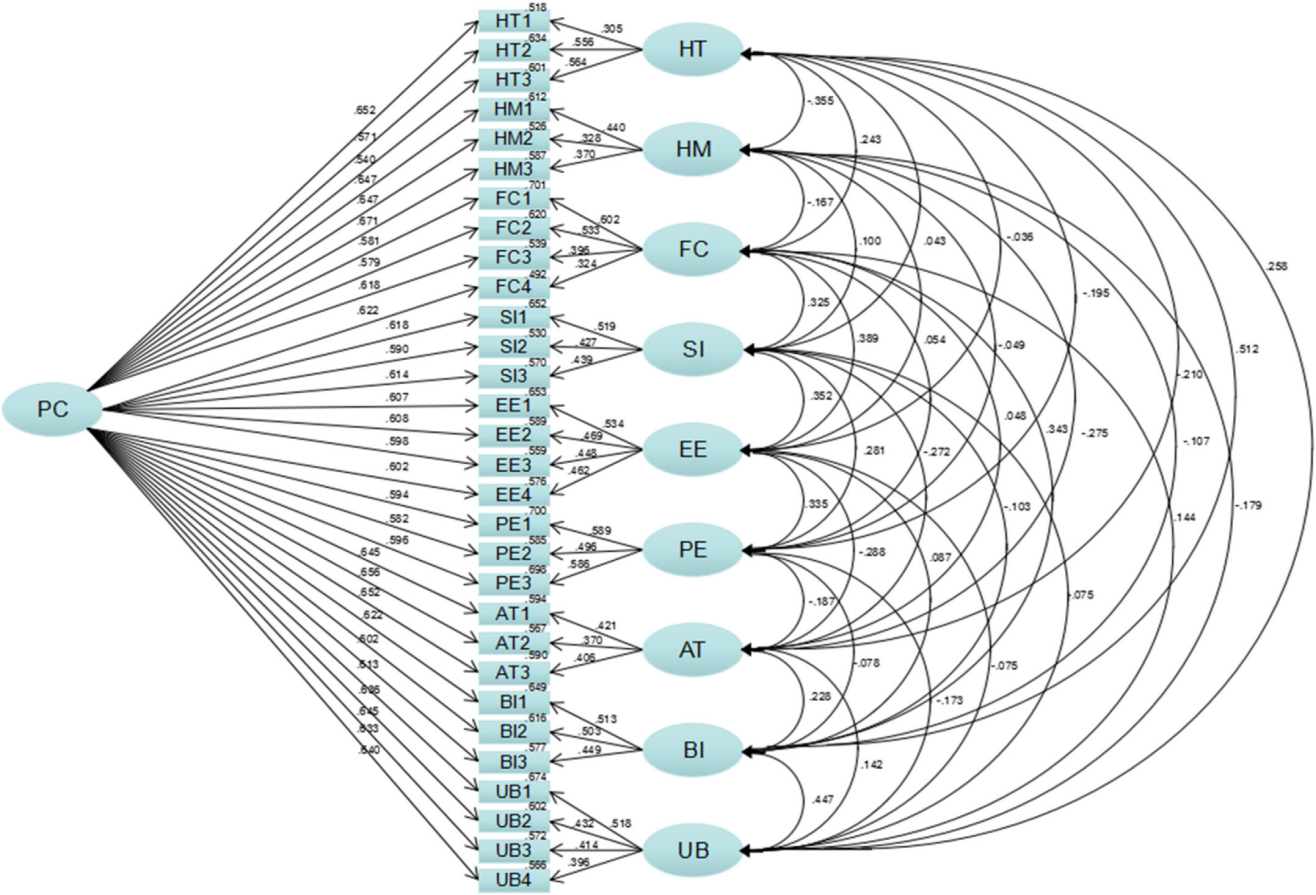
The common method bias model.

In Figure 1, all latent variables are relevant and satisfy path analysis. Based on Table 8, the fit index falls within the range of the recommended values. In comparison to the CFA bias fit index, the common method bias fit index did not improve significantly. The decrease of RMSEA and SRMR is less than 0.05, and the increase of GFI, AGFI, CFI, NFI, TLI is less than 0.1. This indicates that there are no issues related to common method bias.

**Table 8:** Testing for common method bias.

Common Indices	χ2	df	χ2/df	RMSEA	GFI	AGFI	CFI	NFI	TLI	SRMR
Judgement criteria			<3	<0.08	>0.9	>0.9	>0.9	>0.9	>0.9	<0.08
Value	721.481	368	1.961	.030	.957	.946	.980	.961	.977	.025

#### 4.5.2 The first order confirmatory factor analysis

**Figure 2:**
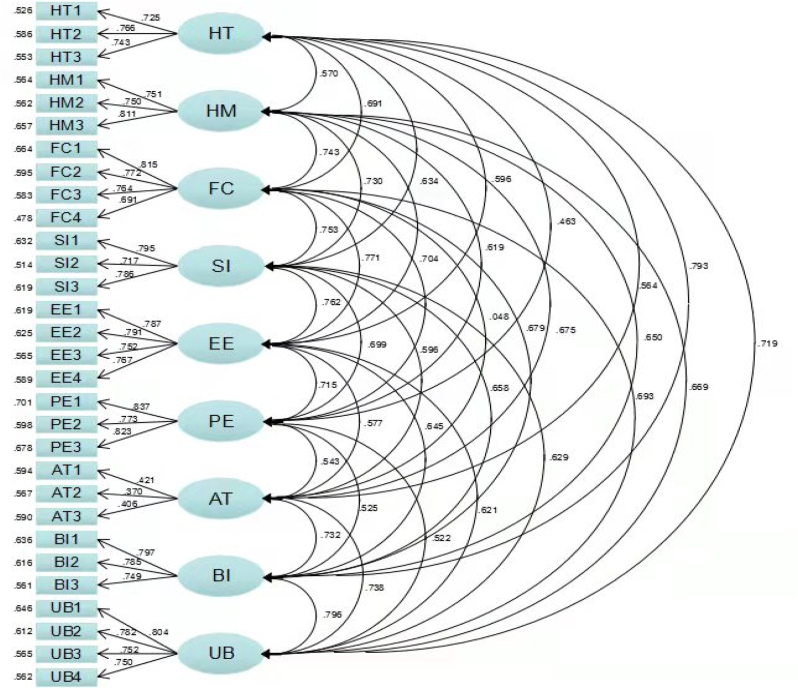
First-order CFA model.

In Figure 2 shows the First-order CFA model. Table 9 shows that the model fitting indicators are in accordance with the recommended indications. CFI = 0.975, RMSEA = 0.034, indicating that the fitting effect is very good (Baik et al., 2020). This indicates that the first-order CFA model has a better fit to the data.

**Table 9:** Adaptation indices of the first-order CFA model.

Common Indices	χ2	df	χ2/df	RMSEA	GFI	AGFI	CFI	NFI	TLI	SRMR
Judgement criteria			<3	<0.08	>0.9	>0.9	>0.9	>0.9	>0.9	<0.08
Value	822.412	369	2.229	.034	.952	.939	.975	.955	.970	.029

#### 4.5.3 Results of the structural equation models

**Figure 3:**
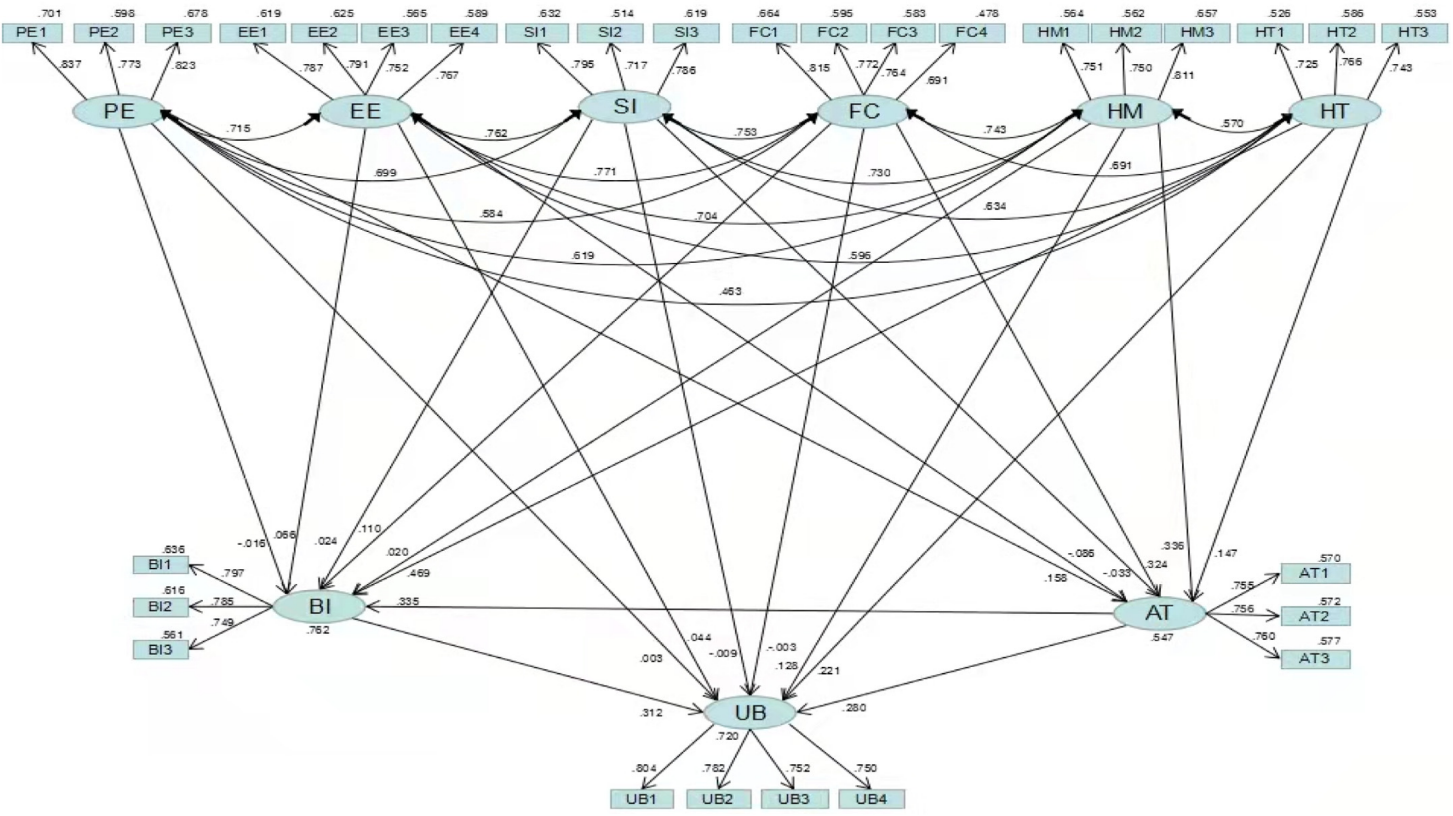
Structural equation model.

In Figure 3, the model path relationship can be seen. Based on Table 10, it can be seen that the model fitting indicators are higher than the recommended standard values (CFI = 0.975, RMSEA = 0.034). This indicates that the model has been properly constructed and that the standard fit has been achieved (Fornell and Larcker, 1981b).

**Table 10:** Adaptability of SEM.

Common Indices	χ2	df	χ2/df	RMSEA	GFI	AGFI	CFI	NFI	TLI	SRMR
Judgement criteria			<3	<0.08	>0.9	>0.9	>0.9	>0.9	>0.9	<0.08
Value	822.412	369	2.229	.034	.952	.939	.975	.955	.970	.029

In this study, the direct and indirect effects of each facets path are estimated using maximum likelihood estimation. In our study, several antecedents were found to have a statistically significant relationship with behavioral intention. The results in Table 11 show that behavioural intention is significantly predicted by HT (ß = .469, P = .002) and AT (ß = .335, P = .001), indicating that Habit and Attitude have a metaverse for college students. There is a positive causal relationship between behavioural intention and technical learning basketball. Nevertheless, social influence (ß = .024, P = .752) and effort expectancy (ß = .066, P = .485) did not confirm a significant route relationship between behavioural intention and metaverse technology, thus concluding that social influence and effort expectancy have no significant effect on the behavioural intention of college students using metaverse technology to learn basketball. Furthermore, HM(ß = .113,P = .001)、FC(ß = .109,P = .007)、PE (ß = .053,P = .032) has an indirect and significant influence on behavioural intention of college students using metaverse technology, indicating that hedonic motivation, facilitating conditions, and performance expectancy are mediator variables.

**Table 11:** Direct and indirect effects.

	Direct effect	Sig.	Indirect effect	Sig.	Total effect	Sig.
	Β		Β		β	
HT→AT	.147	.079	/	/	.147	.079
HT→BI	.469	.002	.049	.047	.518	.003
HT→UB	.221	.050	.203	.006	.424	.001
HM→AT	.336	.001	/	/	.336	.001
HM→BI	.020	.752	.113	.001	.133	.115
HM→UB	.128	.161	.136	.005	.263	.004
FC→AT	.324	.011	/	/	.324	.011
FC→BI	.110	.330	.109	.007	.219	.066
FC→UB	**−**.003	.982	.159	.012	.156	.131
SI→AT	**−**.033	.804	/	/	**−**.033	.804
SI→BI	.024	.752	**−**.011	.766	.013	.884
SI→UB	**−**.009	.886	**−**.005	.974	**−**.014	.876
EE→AT	**−**.086	.393	/	/	**−**.086	.393
EE→BI	.066	.485	**−**.029	.358	.038	.712
EE→UB	.044	.711	**−**.012	.796	.032	.721
PE→AT	.158	.046	/	/	.158	.046
PE→BI	**−**.016	.824	.053	.032	.037	.576
PE→UB	.003	.924	.056	.123	.059	.443
AT→BI	.335	.001	/	/	.335	.001
AT→UB	.280	.003	.105	.017	.384	.002
BI→UB	.312	.025	/	/	.312	.025

In this study, use behavior was significantly related to HT (ß = .221, P = .050), AT (ß = .280, P = .003) and BI (ß = .312, P = .025) Path relationship. This indicates that habit, Attitude, behavioural Intention have a positive causal relationship to the use behavior of college students using metaverse technology to learn basketball. HM (ß = .136, P = .005) and FC (ß = .159, P = .012) have indirect and significant effects on user behavior. Indicating that in college students using metaverse technology to learn basketball, hedonic motivation and facilitating conditions are mediating variables. However, SI (ß = **−**.009, P = .886), EE (ß = .044, P = .711), and PE (ß = .003, P = .924), have been confirmed to have no significant route relationship between the use behavior of college students using metaverse technology.

It has been demonstrated that the factors of UTAUT2, HM (ß = .336, P = .001), FC (ß = .324, P = .011), and PE (ß = .158, P = . 046) have a direct and significant predictive effect on attitude. The results of this study indicate that hedonic motivation, facilitator conditions, and performance expectancy have a positive effect on college students' attitudes toward learning basketball using metaverse technology. Additionally, HT (ß = .147, P = .079), SI (ß = **−**.033, P = .804), and EE (ß = **−**.086, P = .393) use metaverse technology attitudes have no significant effect.

According to Table 12, a new competition model A has been added to HT→BI、HT→UB、HM→AT、FC→AT、PE→AT、AT→BI、AT→UB、BI→UB, and the null hypothesis (β1 = β2)has been established. To test the moderating effect of the competition model A, compare it with the initial model.

**Table 12:** Moderating effect results.

	Original model				Specify β1 = β2 model			
	boy	girl	FR	SO	boy	girl	FR	SO
HT→BI	.469	.433	.566	.317	.468	.434	.504	.478
HT→UB	.046	.324	.134	.221	.258	.283	.160	.194
HM→AT	.279	.361	.428	.181	.286	.359	.344	.357
FC→AT	.554	.193	.280	.429	.327	.314	.346	.365
PE→AT	.150	.166	.164	.079	.177	.151	.122	.149
AT→BI	.503	.321	.416	.259	.381	.348	.392	.323
AT→UB	.190	.331	.331	.292	.287	.319	.322	.308
BI→UB	.443	.191	.313	.354	.209	.238	.312	.354

The results of Table 13 indicate that gender in both the competition model A and the initial model has a significant moderating effect on the path relationship between FC→AT and FC. In the path relationship between HT→BI, HM→AT, grade has a significant moderating effect. There is no significant moderating effect of gender on HT→BI, HT→UB, HM→AT, PE→AT, AT→BI, AT→UB, BI→UB pathways. In addition, the adjustment effect of grade on the HT→UB, FC→AT, PE→AT, AT→BI, AT→UB, BI→UB paths is not significant. According to this study, the habit of using technology to learn basketball has a moderating effect on behavioural intentions and hedonic motivation, and gender has a moderating effect on facilitating conditions.

**Table 13:** Results of the adjustment effect test.

	Gender		Grade	
	CMIN	P	CMIN	P
HT→BI	.000	.983	7.337	.007
HT→UB	3.161	.075	.217	.641
HM→AT	.006	.939	4.416	.036
FC→AT	5.817	.016	.845	.358
PE→AT	.147	.702	1.037	.309
AT→BI	1.810	.179	.923	.337
AT→UB	.517	.472	.051	.821
BI→UB	2.438	.118	.000	.999

## 5 Discussions

UTAUT2 and educational validation were used in this study to examine college students' willingness to use metaverse technology in order to learn basketball. Verified the role of gender and grade in the analysis of several explanatory variables from PE, EE, SI, FC, HM, HT, BI, UB, and AT. Using gender and grade as moderator variables, we can analyze the relationship between college students' willingness to use metaverse technology to learn basketball and other variables. Moreover, the findings of this paper contribute new information to the existing literature and provide references for integrating metaverse technology into physical education programs.

RMSEA, GFI, AGFI, and CFI are all within the threshold range when compared to the fitting indicators of the structural model and the model itself. Clearly, 9 variables, PE, EE, SI, FC, HM, HT, BI, UB, AT, meet the reliability standard of the structure, have high internal consistency, and the Cronbach's Alpha value is greater than 0.787, while AVE values are acceptable (greater than 0.555).

The hypothesis H6 is valid, which shows that Habit has a positive causal relationship with behavioural intention among college students using metaverse technology to learn basketball. Based on the data processing results, habit has the greatest influence coefficient on behavioural intention, meaning habit is an important factor for college students using metaverse technology to learn basketball behavioral intentions. It is the same result as reported by Ramírez-Correa et al., 2019. In our study, habit and behavioural intention are important relationships, as proposed by Hew et al. (2015). Thus, the subject's habit of using metaverse technology to learn basketball is an important variable that affects its behavior. Due to the disadvantages of traditional learning, students realize that the new method is more conducive to improving their academic performance. Metaverse technology has obvious advantages over traditional teaching and facilitates the formation of a habit.

H2, H3 is invalid. In the study, the results indicate effort expectancy and social influence do not significantly affect the behavioral intentions of college students using metaverse technology to learn basketball. It may be related to the purpose of our mission. Faqih & Jaradat believe that it is an easy process (Faqih and Jaradat, 2021). A college student's acceptance of high-tech is generally better than that of a professional. Effort expectancy influences people's behavior less, which supports the findings of S. W. Lee, Sung, & Jeon (Lee et al., 2019, Lew et al., 2019). Despite the fact that social influence is not a decisive factor in this study (Hew et al., 2015), also maintain the viewpoint that basketball learning in college is dominated by students, which is a form of personal behavior, and they are influenced by others and others when choosing a learning method voluntarily. In the context of rewarding or punishing behavior, people are more likely to achieve the expectations of others (Venkatesh et al., 2003).

H1, H4, H5 is invalid. There is a mediating relationship between performance expectancy, facilitating conditions, and hedonic motivation for college students to use metaverse skills. College students should also consider hedonic motivation, facilitating conditions, and performance expectancy when using metaverse technology to learn basketball. This study aims to utilize metaverse technology for teaching, improving academic performance, and enhancing learning outcomes. As the current metaverse technology is implemented as games, performance expectancy and hedonic motivation may have a confounding effect on use intentions during use. A second factor is that metaverse technology is still in development, and the product is not yet ready for purchase and widespread use. The school may be able to provide some assistance, but it is also one of the factors that students consider when planning to use the service frequently.

H6a is valid. Habit has a positive effect on how college students use metaverse technology to learn basketball. The behavior of subjects when learning basketball with habit using metaverse technology is also confirmed by previous finding (J. Lee, Lee et al., 2015; Alalwan et al., 2018; Yuan et al., 2015). In our opinion, the habit here is the basketball habit for college students. In the metaverse, students learn basketball lessons as if they were performing their daily activities, and gradually develop habitual behavior that promotes the use of metaverse technology.

H1a, H2a, H3a is invalid. In college students who use metaverse technology to learn basketball, performance expectancy, effort expectancy, and social influence do not make a significant difference in their use behavior. Subjects' effort expectancy and performance expectancy do not influence their use behavior, which may be due to the fact that the metaverse technology is a new type of technology, and the imaginary hypothetical environment is difficult for unknown technologies to operate in (Schmitz, D'Az-Mart'n, & Guillén, 2022). Shares some similarities with this discovery. H4a, H5a is invalid. It has been found that facilitating conditions and hedonic motivation are mediating variables for the use behaviors of college students using metaverse technology to learn basketball, which differs from previous research findings. Migliore, Wagner, Cechella, & Liébana-Cabanillas contend that FC and HM have no effect on UB (Migliore et al., 2022). Students will learn basketball skills better if they are provided with facilitating conditions for learning using metaverse technology and equipment. In addition, designers and educators should carefully consider the appropriate setting and clear division of their educational and entertainment content.

A fascinating finding in our results is that three variables in the UTAUT2 model are significantly correlated with our newly added variable attitude (AT). H1b, H4b, H5b is valid. A positive causal relationship exists between performance expectations, facilitating conditions, and hedonic motivation, with HM having the greatest impact on college students' attitudes towards using metaverse technology to learn basketball. The performance expectancy, effort expectancy, and social influence of pre-service teachers using learning management systems (LMS) are associated with attitudes, but facilitating conditions do not influence attitudes (Buabeng-Andoh and Baah, 2020). Compared to traditional classroom participants who believe that metaverse technology is better, or that using metaverse technology with the assistance of others is more conducive to learning, a certain technical background can also encourage students to use new technology. There is a positive correlation between consumer hedonic motivation and attitude (Won and Kim, 2020). Students find metaverse technology to be useful, enjoyable, and have a positive attitude toward using it.

H2b, H3b, H6b is invalid. There is no obvious statistical relationship between effort expectancy (EE), social influence (SI), habit (HT) and attitude (AT). The results of these studies are in contrast to those of studies (Dwivedi et al., 2019) that provide evidence that technical ability may have an influence on individual attitudes, and that individuals may also improve their attitudes as a result of information shared by others. Habit is viewed as an unimportant aspect of behavior, and it is analyzed from different perspectives by Automaticity (2016). Several participants have less implementation of metaverse technology, are temporarily unable to form automation, and are not impacted by attitudes. Since metaverse technology is in the developmental stage, the subjects have not really felt the convenience of using the technology, so the attitude has not been impacted.

H7 is valid. It has been demonstrated that the behavior intentions of college students who learn basketball using metaverse technology are positively related to their use behavior and behavioural intention. In this study, we supplement the work of Kondrat (2017), who found that behavioural intention has a significant impact on use behavior and that participants are only likely to engage in use behavior if they are willing to utilize metaverse technology to teach basketball.

H8 is valid. College students who use metaverse technology to learn basketball have a positive causal relationship with behavioral intentions. It confirms previous findings (Ibrahim et al., 2021; Q. Jiang, J. Sun, C. Yang, & C. J. S. Gu, 2022; Lee et al., 2021, Lee et al., 2021). Their study suggests that attitude plays a significant and direct role in behavioural intention. They emphasize that positive attitudes are necessary to improve the behavioural intention of college students using metaverse technology.

H8a is valid. There is a positive causal relationship between the attitude of college students using metaverse technology to learn basketball and their use behavior, and a number of studies have confirmed this conclusion (Cheung and To, 2019) (Amoako et al., 2020). The use behavior of people is determined by their attitude. In addition, performance expectancy, facilitating conditions, and hedonic motivation have an impact on college students' attitudes towards metaverse technology, and attitudes have an impact on college students' behavioral willingness and use of metaverse technology.

H5d, H6d is valid. In terms of grade, habit has only a moderating effect on behavioural intention and hedonic motivation, both of which are more significant for freshmen than sophomores. It may be the case that sophomores tend to rely on their original habit (Venkatesh et al., 2012). suggested that older people are more likely to rely on habit as a result of their experience. Freshmen are more receptive to new experiences when they enter college. Learning methods are more appealing to them, they feel more joy and fun when playing games, and they view metaverse technology as more enjoyable and fun. Using metaverse technology, we can encourage them to learn basketball and show a positive attitude.

H4c is valid. Gender moderates attitudes regarding the facilitation of basketball lessons in the metaverse by college students. It differs from previous findings (Nikolopoulou et al., 2020). According to our results, men are more likely than women to be affected by facilitating conditions. To some extent, basketball was a sport primarily preferred by boys, with a higher frequency and higher number of participants. They are more inclined to learn basketball in novel ways because of their passion for the game. Compared to traditional teaching methods, boys find metaverse technology more convenient for learning basketball lessons, and this is reflected in their positive attitudes. It is less appealing to girls who are less enthusiastic about basketball and require less training.

### 5.1 Theoretical contributions

First, this study contributes to the existing literature on metaverse technology in several ways. Although there is a great deal of research being conducted on the metaverse, there is still a lack of research in its application to sports. Therefore, this research contributes to the understanding of metaverse technology in sports on a practical level. It provides a reference for future metaverse and sports research as well as enriches existing research.

A further academic contribution of this paper is the use of the UTAUT2 model. Based on a review of the previous literature, UTAUT2 is widely used to study technology acceptance and can be used to explain the content of this article more effectively than other models. In this regard, is selected to assume the theoretical basis of the model. Using the UTAUT2 theory, attitude variables are added to the variables in the UTAUT2 model in order to assess college students' intentions to use metaverse technology to learn basketball courses. The model is expanded to include attitude variables and adapted to fit the research focus. The study hypothesizes causal relationships between variables, identifying the most influential factors that affect participants' willingness to use and their behavior in using metaverse data, thereby enhancing our understanding of metaverse data in a more comprehensive manner.

### 5.2 Management contributions

The advancement of science and technology has brought great convenience to people's lives, and metaverse technology exists as a new type of technological innovation. In the context of evolving technologies, the results of this study can assist teachers and students in learning basketball lessons more effectively. It is likely to reduce the impact of changes in the environment if people are able to successfully complete real-world work in a twinned virtual space. A combination of metaverse technology can enhance the student experience, in accordance with the internal perception of students.

This study has made the following contributions to management:

In the first place, we conclude that habit plays an important role. In addition to affecting the way college students use the metaverse to learn basketball, habit plays an important role in influencing their behaviour intentions as well. Through the use of metaverse technology, college students will unconsciously form automatic movements and habits when using the metaverse to learn basketball courses. Thus, it has the potential to substantially improve the utilization of metaverse technology, as well as to promote long-term development of interactive virtual reality teaching. By using metaverse technology in their basketball lessons, there is a greater possibility that they will become more willing to learn, that they will continue to use metaverse technology for lessons, and that they will be able to learn even when they are not confined by space.

Second, the results indicate that attitude is another key variable, and attitude plays a significant role in both use behavior and behavior intention of college students using the metaverse to learn basketball. A student's positive attitude towards learning basketball with meta-meta technology not only enhances the learning effect, but also encourages them to use meta-meta in other courses and for reference. Moreover, hedonic motivation, facilitating conditions, and performance expectancy have a significant impact on college students' attitudes toward learning basketball through metaverse technology. By utilizing metaworld technology in basketball teaching, students will experience a more interactive learning environment and be more motivated to learn. A timely and convenient Internet resource sharing program encourages students to demonstrate positive expectations and awareness. As a result, all of these elements will indirectly encourage the students to continue their education using metaverse technology. Therefore, it has significance for promoting meta-technical teaching in other disciplines.

## 6 Conclusions and recommendations

It is likely that the development of metaverse technology will be the dominant trend in future Internet development as society continues to advance. In this study, we examine the factors that influence college students' willingness to use metaverse technology for basketball lessons. As a result of the current research, a substantial amount of knowledge is added regarding the willingness of college students to use metaverse technology to learn the combination of basketball curriculum willingness and technology acceptance model. This research represents one of the leading research on the use of metaverse technology in sports fields. It should be noted, however, that owing to the online nature of metaverse basketball, there may be some management problems in classrooms. The technology is heavily focused on representation, and the infrastructure is not yet perfect, so a reasonable amount of time will be required for it to be fully implemented.

This paper examines college students' willingness to use metaverse technology to study basketball courses, and the factors that may affect their use of metaverse technology. In order to achieve the above purpose, the conceptual model in this paper is based on the UTAUT2 model. By reducing the price variable and expanding the attitude variable, a new causal path relationship is obtained. Making suggestions for improving the teaching methods of the course by combining physical education with metaverse technology in the future.

This study's statistical results support the hypothesized model's predictive validity. Specifically, habit, attitude, and behavioral intention have been found to be significant predictors of usage behavior. Habit and attitude are also considered important factors for behavioral intention. The factors of hedonistic motivation, facilitating conditions, and performance expectation have a significant impact on attitudes. It was verified in this study that attitude is an important variable and that it is the main factor predicting the use behavior and behavioral intentions of college students who use metaverse technology to learn basketball, and the results contribute to a better understanding of UTAUT2.

Additionally, gender and grade are used as moderators in this study. In this study, it was found that grade has a moderating effect on habit and behavioural intention. Hedonic motivation has a moderating effect on attitude. Gender has a moderating effect on facilitating conditions in the attitude path. Practitioners can benefit from current research.

## 7 Limitations

Limitations of this study include:

In this study, the first objective is to collect the required data from freshmen and sophomores in college. The current study indicates that the rates for females are significantly higher than those for males, and the subjects may not be representative of the general population. Furthermore, the objective of the research is to determine whether college students are willing to use metaverse technology to learn basketball. As the study was conducted on highly educated students with sufficient Internet experience, it is worthwhile to consider whether the findings are applicable to other groups with different characteristics. Additionally, it may be necessary to study the data collected over a fixed period of time again in order to determine their long-term relevance. The development of metaverse technology is still in its infancy, and the future direction and specific operation methods are still in the exploratory stages.

## 8 Further research

A future study could include students of other grades and the research content could be investigated over a wide range of time periods. Data collection in terms of boys and girls is relatively balanced, thus making the characteristics of the research objects more comprehensive. It is possible to expand the metaverse technology by combining it with other sports and courses, as well as from other perspectives. Utilization of virtual reality educational tools such as virtual reality, artificial intelligence, game apps, and metaverse equipment (such as virtual reality gloves, augmented reality glasses, etc.). The research results can be used to further explain the willingness of college students to use metaverse technology for basketball courses, as well as empirical studies on the improvement of knowledge and skills about metaverse technology in the future. Our team will continue to monitor the development of metaverse technologies and equipment, hoping that metaverse can offer a comprehensive application of concepts and technologies to the field of education.

## Declarations

### Author contribution statement

Fangfang Yang: Performed the experiments; Wrote the paper.

Longfei Ren: Conceived and designed the experiments; Wrote the paper.

Chao Gu: Analyzed and interpreted the data; Contributed reagents, materials, analysis tools or data.

### Funding statement

This research did not receive any specific grant from funding agencies in the public, commercial, or not-for-profit sectors.

### Data availability statement

Data will be made available on request.

### Declaration of interest’s statement

The authors declare no conflict of interest.

### Additional information

No additional information is available for this paper.

## Appendix A Supplementary data

Questionnaire Questionnaire
