# Supplementary material for: A study of college students' intention to use metaverse technology for basketball learning based on UTAUT2
Source: Heliyon. 2022 Sep 8;8(9):e10562. doi: 10.1016/j.heliyon.2022.e10562 (PMC9483595; doi:10.1016/j.heliyon.2022.e10562)
Supplement: Questionnaire [file mmc1.docx]

Questionnaire

| LatentVariable | Coding | Item | Source |
| --- | --- | --- | --- |
| Performance Expectancy | PE1 | I found it useful to use mateverse techniques in my basketball learning process. | 【(Venkatesh et al., 2012)】 |
|  | PE2 | Using mateverse technology can help me finish my basketball study faster. |  |
|  | PE3 | Using mateverse technology can improve my efficiency in learning basketball. |  |
| Effort Expectancy | EE1 | Learning how to use mateverse technology for basketball learning was easy for me. |  |
|  | EE2 | My interaction with the mateverse technology used to learn basketball is clear and understandable. |  |
|  | EE3 | I find basketball learning through mateverse technology easy to use. |  |
|  | EE4 | It was easy for me to learn basketball using mateverse techniques proficiently. |  |
| Social Influence | SI1 | The people who matter to me think I should use mateverse technology to learn basketball. |  |
|  | SI2 | People who influence my behavior think I should use mateverse technology to learn basketball. |  |
|  | SI3 | The people I care about prefer me to use mateverse technology to learn basketball. |  |
| Facilitating Conditions | FC1 | I have the resources I need to learn basketball using mateverse technology. |  |
|  | FC2 | I have the knowledge required to use mateverse techniques for basketball learning. |  |
|  | FC3 | The mateverse technology used to learn basketball is compatible with other technologies I use. |  |
|  | FC4 | There are people I can turn to for help when I have trouble using the mateverse technology used for my studies. |  |
| Hedonic Motivation | HM1 | Learning basketball using mateverse technology is fun. |  |
|  | HM2 | Learning basketball using mateverse technology is a joy. |  |
|  | HM3 | Learning basketball using mateverse technology is very enjoyable. |  |
| Habit | HT1 | Using mateverse technology for basketball learning will become a habit of mine. |  |
|  | HT2 | I was obsessed with using mateverse technology for basketball learning. |  |
|  | HT3 | I had to use mateverse technology to learn basketball. |  |
| Behavioral Intention | BI1 | I plan to continue to use mateverse techniques for basketball learning in the future. |  |
|  | BI2 | In my daily life I will always try to use mateverse technology for basketball learning. |  |
|  | BI3 | I intend to continue to use mateverse technology for basketball learning on a regular basis. | 【(S. M. Lee & Lee, 2020)】 |
| use behavior | AC1 | I like to use mateverse technology for basketball learning. |  |
|  | AC2 | I will actively use mateverse technology for basketball learning. |  |
|  | AC3 | I would recommend the use of mateverse techniques for basketball learning to those around me. |  |
|  | AC4 | I am confident in using mateverse technology for basketball learning. |  |
| Attitude | AT1 | If I had the opportunity to use the mateverse techniques for basketball learning, I would have a positive view of the metaverse techniques. | 【(Q. Jiang, J. Sun, C. Yang, & C. Gu, 2022)】 |
|  | AT2 | Mateverse technology for basketball learning provides a valuable service. |  |
|  | AT3 | Learning basketball using mateverse technology can be an enjoyable experience. |  |
